# Biomarker-Guided Dietary Supplementation: A Narrative Review of Precision in Personalized Nutrition

**DOI:** 10.3390/nu16234033

**Published:** 2024-11-25

**Authors:** Evgeny Pokushalov, Andrey Ponomarenko, Evgenya Shrainer, Dmitry Kudlay, Richard Miller

**Affiliations:** 1 Center for New Medical Technologies, Novosibirsk 630090, Russia; ponomarenko_av@cnmt.ru (A.P.); shrayner_ev@cnmt.ru (E.S.); 2 Scientific Research Laboratory, Triangel Scientific, San Francisco, CA 94101, USA; info@triangelcompany.com; 3 Institute of Pharmacy, I.M. Sechenov First Moscow State Medical University (Sechenov University), Moscow 119435, Russia; d624254@gmail.com

**Keywords:** dietary supplements, biomarkers, personalized nutrition, artificial intelligence, precision medicine, targeted supplementation

## Abstract

**Background:** Dietary supplements (DS) are widely used to address nutritional deficiencies and promote health, yet their indiscriminate use often leads to reduced efficacy, adverse effects, and safety concerns. Biomarker-driven approaches have emerged as a promising strategy to optimize DS prescriptions, ensuring precision and reducing risks associated with generic recommendations. **Methods:** This narrative review synthesizes findings from key studies on biomarker-guided dietary supplementation and the integration of artificial intelligence (AI) in biomarker analysis. Key biomarker categories—genomic, proteomic, metabolomic, lipidomic, microbiome, and immunological—were reviewed, alongside AI applications for interpreting these biomarkers and tailoring supplement prescriptions. **Results:** Biomarkers enable the identification of deficiencies, metabolic imbalances, and disease predispositions, supporting targeted and safe DS use. For example, genomic markers like MTHFR polymorphisms inform folate supplementation needs, while metabolomic markers such as glucose and insulin levels guide interventions in metabolic disorders. AI-driven tools streamline biomarker interpretation, optimize supplement selection, and enhance therapeutic outcomes by accounting for complex biomarker interactions and individual needs. **Limitations:** Despite these advancements, AI tools face significant challenges, including reliance on incomplete training datasets and a limited number of clinically validated algorithms. Additionally, most current research focuses on clinical populations, limiting generalizability to healthier populations. Long-term studies remain scarce, raising questions about the sustained efficacy and safety of biomarker-guided supplementation. Regulatory ambiguity further complicates the classification of supplements, especially when combinations exhibit pharmaceutical-like effects. **Conclusions:** Biomarker-guided DS prescription, augmented by AI, represents a cornerstone of personalized nutrition. While offering significant potential for precision and efficacy, advancing these strategies requires addressing challenges such as incomplete AI data, regulatory uncertainties, and the lack of long-term studies. By overcoming these obstacles, clinicians can better meet individual health needs, prevent diseases, and integrate precision nutrition into routine care.

## 1. Introduction

In recent decades, dietary supplements (DS) have gained widespread popularity worldwide, used to address nutritional deficiencies, support health, and prevent diseases. Dietary supplements are defined as products intended to supplement the diet, containing one or more ingredients such as vitamins, minerals, herbs, or other botanicals. According to the US Food and Drug Administration (FDA), dietary supplements are distinct from prescription drugs, traditional herbal medicines, and alternative therapies, as they are not intended to treat, diagnose, prevent, or cure diseases [1]. Products making such claims fall under the category of drugs and are subject to stricter regulatory requirements.

Dietary supplements have become an integral part of the lifestyle for people across different age groups. It is estimated that over half of the adult population in developed countries regularly uses various supplements, including vitamins, minerals, antioxidants, and other active compounds [2]. However, the widespread and often unsupervised use of DS raises significant concerns within the scientific and medical communities. Studies indicate that uncontrolled use of supplements may not align with actual clinical needs and, in some cases, may result in adverse effects and complications, such as heavy metal toxicity and unfavorable drug interactions [3,4]. These risks are particularly pronounced with supplements that lack proper regulatory approval or fail to meet quality certification standards, highlighting the importance of selecting products that comply with established safety regulations.

Additionally, while healthcare professionals may recommend dietary supplements based on individual needs and biomarker analysis, these products are often self-prescribed by consumers. This practice increases the risk of improper use, excessive dosages, and adverse outcomes, underscoring the necessity for informed guidance and monitoring in the use of dietary supplements.

Systematic prescription of DS without accounting for individual needs and without biomarker analysis often diminishes their efficacy and undermines DS as a preventive health tool. In the absence of a personalized approach, supplements are frequently perceived as “one-size-fits-all” solutions, despite research indicating that indiscriminate use may be not only ineffective but also potentially harmful [5]. A comprehensive approach based on biomarker data allows for accurate identification of nutritional deficiencies and metabolic requirements, thus improving both the precision and safety of DS prescription [6,7]. Additionally, the integration of artificial intelligence (AI) for analyzing biomarker data can further assist clinicians and nutritionists in optimizing dosages and combinations of supplements according to individual patient needs [8].

### 1.1. Aim of the Review

This review aims to provide a narrative overview of the potential of biomarker-guided dietary supplementation to enhance the precision and efficacy of personalized nutrition. By synthesizing current knowledge, the review highlights how biomarkers and AI-driven methods can enable healthcare providers to make more informed and effective dietary supplement recommendations.

### 1.2. Objectives of the Review

To address the aim, this narrative review focuses on:

- Evaluating the role of biomarkers in personalized nutrition: Identifying key biomarker categories and their significance in guiding dietary supplementation to meet individual health needs.
- Exploring AI-driven approaches: Reviewing how artificial intelligence facilitates the analysis of biomarker data, enabling tailored supplementation strategies for disease prevention and management.
- Assessing the risks of uncontrolled dietary supplement use: Discussing the dangers of indiscriminate supplementation and emphasizing the importance of biomarker-driven precision to improve safety and effectiveness.
- Providing actionable insights for healthcare providers: Offering practical guidelines for implementing biomarker-based and AI-enhanced dietary supplement strategies in clinical practice.

## 2. Risks and Limitations of Uncontrolled Use of Dietary Supplements

The uncontrolled use of dietary supplements has become a significant healthcare issue, often based on generic recommendations that overlook individual health needs. The easy availability of these products and aggressive marketing encourage self-prescription without clinical indications, potentially leading to low efficacy and adverse health effects.

The traditional, one-size-fits-all approach to DS use disregards the unique biochemical and physiological characteristics of each patient, reducing the effectiveness of such supplements. Generic recommendations are often not only ineffective but can also harm the body. For example, iron supplements are frequently recommended without assessing iron levels in the body, which can lead to iron accumulation, increasing cardiovascular risks and liver damage [9,10]. Antioxidants such as vitamin E and β-carotene have been shown to increase mortality among individuals taking them for chronic disease prevention [11,12,13]. Meta-analyses further indicate that taking multivitamin complexes and antioxidants does not reduce cardiovascular disease risk or improve overall survival in adults with normal nutrient levels [5,11,12,13]. Studies also show that vitamin C and E intake does not reduce the risk of colds in healthy individuals, questioning their prophylactic use in the general population [14,15]. Therefore, universal DS recommendations may be not only ineffective but also contribute to side effects and reduce confidence in these products.

Uncontrolled DS intake may also cause side effects, such as hypervitaminosis, and dangerous interactions with medications [16,17,18]. Taking fat-soluble vitamins like A and D without medical supervision can lead to accumulation in the body and toxicity [19,20]. Excessive levels of vitamin D, for instance, may cause hypercalcemia, resulting in vascular calcification and organ damage [20]. Vitamin E can reduce the effectiveness of chemotherapy in cancer patients, protecting both healthy and cancerous cells [17]. Omega-3 fatty acids increase bleeding risk in patients taking anticoagulants, and St. John’s Wort can weaken the effects of antidepressants and HIV medications, potentially worsening the patient’s condition [21,22]. Magnesium can also decrease the efficacy of certain antibiotics, such as tetracyclines and fluoroquinolones, complicating the treatment of infections [23]. Excess calcium intake has been associated with an increased risk of cardiovascular diseases. A meta-analysis showed that individuals taking high-dose calcium supplements may face an increased risk of atherosclerosis and arterial calcification, raising the likelihood of heart attacks and strokes [24].

The low quality control of some supplements poses an additional health risk. Studies indicate that certain DS products may contain heavy metal contaminants, such as lead, mercury, and cadmium, which can lead to toxic damage to the liver, kidneys, and nervous system when consumed over time [25]. Additionally, some weight loss or energy-enhancing supplements contain hazardous undeclared ingredients, such as ephedrine, which heightens the risk of cardiovascular complications, including arrhythmias, hypertension, and strokes [26]. Beyond heavy metals, studies reveal the presence of active pharmaceutical ingredients in some supplements, which pose a risk for patients who do not expect to encounter drug effects. For instance, stimulants found in weight loss supplements can adversely affect the cardiovascular system and cause serious consequences for patients with heart conditions [27].

## 3. Rationale for Using Biomarkers in Targeted Dietary Supplement Prescription

Biomarkers are objective, measurable indicators that enable more precise assessment of health status, identification of deficiencies, and consideration of an individual’s metabolic characteristics. In the context of dietary supplement (DS) prescription, biomarkers help determine the body’s true needs, reducing subjective assessments and supporting scientifically grounded recommendations. Unlike traditional approaches based on general recommendations that overlook individual differences, biomarker analysis allows accurate identification of nutrient deficiencies, facilitating targeted and safe supplementation [28,29,30].

Biomarkers allow healthcare professionals to determine whether a patient genuinely needs a specific supplement, thereby reducing the risk of overuse and potential toxicity. This is particularly important for fat-soluble vitamins, such as A, D, E, and K, which accumulate in the body and can lead to toxic effects when consumed in excess [31]. It is essential to ensure that dietary supplements are approved by regulatory authorities, as unregulated products may contain incorrect dosages or harmful substances, increasing the risks associated with their use. Additionally, biomarkers help prevent unnecessary recommendations, enhancing both the safety and efficacy of DS therapy.

Several categories of biomarkers are widely used to assess specific nutrient requirements in the body, including:

- Genetic (genomic) biomarkers: These biomarkers reveal predispositions to deficiencies and chronic diseases, as well as the ways the body metabolizes certain nutrients. For instance, gene polymorphisms can indicate a need to adjust diet or recommend supplements based on genetic predispositions [32].
- Proteomic biomarkers: These biomarkers measure protein levels related to inflammatory and metabolic processes, enabling physicians to assess nutrient stores (e.g., iron) and levels of inflammation, thus supporting targeted antioxidant and anti-inflammatory DS prescriptions [33].
- Metabolomic biomarkers: Metabolites such as glucose and lipids reflect metabolic status and can help prevent disorders like diabetes and cardiovascular disease. Metabolomic markers are fundamental for recommending DS that improve metabolism and regulate blood sugar and cholesterol levels [34].
- Lipidomic biomarkers: Lipid biomarkers reflect lipid metabolism and are used to assess cardiovascular risk. Lipid profiles allow for the evaluation of DS needs, such as omega-3 fatty acids and niacin, to support cardiovascular health [35].
- Epigenetic biomarkers: These biomarkers show changes in gene expression caused by environmental factors, stress, and diet. They enable the assessment of how specific nutrients might impact epigenetic mechanisms, which is particularly important under chronic stress or prolonged nutrient deficiency [36].
- Microbiome biomarkers: These biomarkers assess gut microbiota composition and help identify imbalances linked to digestive and immune issues. Based on microbiome analysis, physicians can recommend probiotics and prebiotics that support gut health and immune function [37].
- Immunological biomarkers: Immunological biomarkers, such as cytokine levels or immune cell counts, provide insights into immune response and inflammation in the body. These biomarkers aid in selecting supplements that support immunity and have anti-inflammatory effects, such as vitamins D and C, zinc, and omega-3 fatty acids [38].

Thus, the use of biomarkers in medicine enables a personalized approach to DS prescription, focusing strictly on the specific needs of each patient. Biomarkers facilitate improved metabolic balance and reduced risk of chronic diseases, while allowing precise monitoring of treatment efficacy.

## 4. Genetic (Genomic) Biomarkers for Targeted Dietary Supplement Prescription

Genetic biomarkers are based on gene polymorphisms that can indicate a predisposition to certain vitamin or mineral deficiencies, metabolic disorders, and chronic diseases. These polymorphisms influence how the body metabolizes or absorbs specific nutrients [39,40,41].

### 4.1. Key Genes and Their Role in Nutrient Metabolism

There are several key genes that significantly impact nutrient metabolism and are widely used in clinical practice. These genes aid in the personalized selection of supplements and dosages for optimal safety and efficacy.

- MTHFR Gene: This gene is responsible for folate metabolism, influencing levels of the active form of folic acid (5-MTHF) and vitamin B12. Polymorphisms in MTHFR can impair the body’s ability to convert folic acid into its active form, which is critical for cardiovascular and nervous system health. Patients with MTHFR variations are often recommended methylated forms of folates (5-MTHF) and vitamin B12 to prevent deficiency and maintain necessary levels [42].
- APOE Gene: This gene influences lipid metabolism and is associated with cardiovascular disease risk and neurodegenerative disorders, such as Alzheimer’s disease. Patients with certain APOE polymorphisms, especially APOE ε4, are more prone to elevated cholesterol levels and inflammation. For these patients, omega-3 fatty acids and antioxidants are often recommended to help reduce inflammation and maintain a healthy lipid profile [43,44].
- VDR Gene: The vitamin D receptor gene (VDR) controls the activation and absorption of vitamin D, as well as calcium metabolism, which is crucial for bone mass maintenance and osteoporosis prevention. Polymorphisms in VDR may limit the body’s ability to effectively absorb vitamin D, requiring higher doses of this vitamin, along with vitamin K2, to support bone and cardiovascular health [45].
- COMT Gene: This gene regulates the metabolism of catecholamines, including dopamine and adrenaline, and influences stress resilience. COMT polymorphisms can indicate increased sensitivity to stress and reduced capacity for neurotransmitter metabolism. Patients with COMT variations may benefit from supplements with magnesium and B vitamins to support the nervous system and manage stress levels [46,47].

These genes are the most extensively studied and have a significant impact on metabolic processes; however, they represent only a portion of the genetic information that is useful for DS prescription.

### 4.2. Additional Genes for Advanced Nutritional Personalization

In addition to key genes, there are other genes that can be helpful in practical applications, especially in complex cases or when deeper personalization of therapy is needed:

- CYP1A2—This gene encodes an enzyme involved in caffeine and xenobiotic metabolism. Polymorphisms in CYP1A2 influence antioxidant capacity and can guide the optimal prescription of antioxidant supplements [48].
- SOD2—The superoxide dismutase gene plays a role in cellular protection against oxidative stress. Polymorphisms in SOD2 may indicate a higher need for antioxidants, such as vitamins C and E [49].
- FTO—This gene is associated with obesity and metabolic syndrome. FTO polymorphisms may influence susceptibility to obesity and dietary habits, guiding the need for supplements that aid weight management and insulin control [50].
- TNF-α and IL-6—These genes are involved in inflammatory processes. Polymorphisms in TNF-α and IL-6 can increase inflammation susceptibility and may indicate the need for anti-inflammatory supplements such as omega-3 fatty acids and curcumin [51,52].
- ACE and PPARG—These genes regulate blood pressure and lipid metabolism. Their polymorphisms help assess the risk of hypertension and metabolic disorders, guiding the choice of supplements for cardiovascular support [53,54].

These and many other genes help create a more detailed understanding of a patient’s metabolic characteristics and nutrient needs. They allow healthcare providers to account for not only common deficiencies and risks but also specific genetic factors that may influence nutrient absorption, metabolic processes, and chronic disease likelihood. Expanding genetic analysis and integrating it with other biomarkers enables the creation of a multidimensional health profile, supporting a highly personalized approach to DS prescription.

## 5. Proteomic Biomarkers for Targeted Nutraceutical Supplementation

Proteomic biomarkers provide valuable insights into inflammatory processes, immune responses, and nutrient status. These biomarkers allow clinicians to select tailored nutritional interventions that support immune function, modulate inflammation, and optimize metabolic health.

### 5.1. Key Proteomic Biomarkers and Their Role in Supplementation

- Ferritin: Ferritin reflects iron storage but can also increase in chronic inflammation. Elevated ferritin may indicate a need for antioxidant support to control inflammation, whereas low ferritin requires iron supplementation with careful dose management to avoid iron overload [55].
- Albumin: Low albumin levels may indicate chronic inflammation or poor nutritional status. Patients with reduced albumin levels may benefit from amino acid supplements and protein complexes to support protein status and improve nutritional outcomes [56].
- Troponins: Troponins are cardiac markers indicative of myocardial injury. Elevated troponins suggest a need for cardiovascular support, such as Coenzyme Q10 and magnesium supplements, which reduce oxidative stress and support heart function [57].

### 5.2. Additional Proteomic Biomarkers for Inflammatory and Metabolic Assessment

- Haptoglobin: Haptoglobin increases with inflammation and may require antioxidant support, especially in conditions associated with elevated iron levels. Antioxidants such as vitamins C and E can help reduce oxidative stress and cellular damage [58].
- Ceruloplasmin: Involved in copper metabolism, ceruloplasmin levels may rise with inflammation. Elevated ceruloplasmin can indicate a need for antioxidant and nutrient support targeting copper metabolism [59].
- Transferrin: This iron-binding protein helps assess iron reserves and anemia risk. Low iron levels necessitate iron supplementation, while excess levels require careful monitoring and possible iron intake restriction [60].
- Fibrinogen: As a protein involved in coagulation and inflammation, elevated fibrinogen is associated with thrombotic risk and chronic inflammation. Omega-3 fatty acids, curcumin, and anticoagulant support can reduce fibrinogen levels and support vascular health [61,62].

## 6. Metabolomic Biomarkers for Targeted Supplementation

Metabolomic biomarkers are small molecules and metabolites—such as glucose, organic acids, and uric acid—that reflect the body’s current metabolic state. These markers help assess the risk of metabolic diseases (e.g., diabetes and inflammatory disorders) and identify deficiencies that can be corrected through targeted nutritional support. Analyzing metabolomic biomarkers enables healthcare providers to determine which supplements can optimize metabolic health, maintain blood sugar balance, and support other key metabolic functions.

### 6.1. Key Metabolomic Biomarkers and Their Role in Supplement Selection

There are several key metabolomic biomarkers that help identify metabolic imbalances and allow precise adjustment of nutritional needs through supplements.

- Glucose: Glucose is the primary energy source in the body, and its blood level reflects the status of blood sugar regulation. Elevated glucose levels can indicate insulin resistance or type 2 diabetes risk. Patients with high glucose levels may benefit from supplements such as chromium and alpha-lipoic acid, which improve insulin sensitivity and help maintain normal blood sugar levels [63].
- Insulin: Insulin levels, particularly in combination with glucose, help assess insulin sensitivity and insulin resistance risk. Elevated insulin levels can indicate metabolic dysfunction even when glucose is within normal ranges. Patients with high insulin may benefit from supplements that support glucose metabolism, such as alpha-lipoic acid and magnesium [64].
- Homocysteine: This amino acid metabolite reflects levels of B vitamins like B12 and folate. Elevated homocysteine may signal a deficiency in B vitamins and is associated with increased cardiovascular disease risk. Patients with high homocysteine are often recommended methylated forms of folate and B12 supplements [65].
- Cortisol: Blood or saliva cortisol levels help assess stress and adrenal function. Patients with elevated cortisol or chronic stress may benefit from supplements such as magnesium and adaptogens (e.g., Rhodiola) for nervous system support [66].
- Uric Acid: Uric acid, a breakdown product of purines, can accumulate due to metabolic imbalances. High uric acid levels can lead to gout and inflammation. Patients with elevated uric acid may benefit from antioxidants like vitamin C, which lower uric acid and protect against oxidative stress [67].
- Lactate: Lactate is a metabolite of anaerobic glucose metabolism. Elevated lactate levels can indicate hypoxia or mitochondrial dysfunction. Patients with high lactate levels may benefit from supplements like Coenzyme Q10 and carnitine, which support mitochondrial function [68].

### 6.2. Additional Metabolomic Biomarkers for Supplementation Guidance

In addition to the key biomarkers, other metabolites can provide further insights into metabolic health and help refine supplement recommendations:

- Organic Acids (e.g., malonic acid, succinic acid): These intermediates in the Krebs cycle and mitochondrial metabolism indicate mitochondrial function. Elevated levels may reflect mitochondrial dysfunction, where magnesium and B vitamins can support normal mitochondrial metabolism [69].
- Ketone Bodies (e.g., β-hydroxybutyrate): Ketone bodies are fat breakdown products used as an alternative energy source when glucose is low, as in ketogenic diets. Patients relying on ketones for energy may benefit from electrolyte and B vitamin supplements to support metabolism [70].
- Amino Acid Profiles (e.g., glutamine, arginine): Levels of amino acids like arginine and glutamine indicate nitrogen balance, muscle status, and immune health. Arginine and glutamine are essential for tissue healing and immune support, making their supplementation valuable under stress or injury [71].
- Creatinine: Creatinine levels are used to assess kidney function and muscle metabolism. High levels can indicate protein overload or impaired kidney function. Patients with high creatinine may benefit from kidney-supporting and anti-inflammatory supplements [72].
- Serotonin and Dopamine Metabolites (e.g., 5-HIAA for serotonin): These neurotransmitter metabolites help assess stress and mental health. They are useful for guiding nervous system support supplements like B vitamins, magnesium, and adaptogens [73].
- Phosphorus and Electrolytes (e.g., sodium, potassium): Electrolyte levels are essential for evaluating acid-base balance and hydration. Supplements with electrolytes can be beneficial, especially for patients with high physical demands [74].
- Creatine Kinase (CK): CK is an enzyme released during muscle damage. Elevated CK can indicate overuse or inflammation, and amino acids, antioxidants, and anti-inflammatory supplements can support recovery [75].
- Acetylcarnitine: This metabolite is linked to mitochondrial function and fat metabolism. Deficiency in acetylcarnitine may indicate energy metabolism issues, which can be supported by carnitine and Coenzyme Q10 supplements [76].
- S-Adenosylmethionine (SAMe): SAMe is involved in methylation, which affects mood, liver health, and B vitamin metabolism. SAMe levels guide supplementation with methylated B vitamins [77].

## 7. Lipid Biomarkers for Targeted Supplementation

Lipid biomarkers, reflecting lipid metabolism status, are used to assess cardiovascular risk and optimize a patient’s lipid profile. These metrics help identify patients needing lipid metabolism adjustments and allow for the selection of effective nutritional interventions targeting the prevention and management of atherosclerosis and metabolic syndrome.

### 7.1. Key Lipid Biomarkers and Their Role in Supplement Selection

- Total Cholesterol, Low-Density Lipoproteins (LDL), High-Density Lipoproteins (HDL), and Triglycerides: These are primary indicators of cardiovascular risk and metabolic health. Elevated levels of total cholesterol and LDL, along with low HDL and high triglycerides, may signal the presence of atherosclerosis, metabolic syndrome, and other cardiovascular issues. Supplements like omega-3 fatty acids, plant sterols, and niacin are recommended for improving these parameters and reducing cardiovascular risk. Omega-3 fatty acids help to lower total cholesterol and triglycerides, as well as increase HDL, thus reducing atherosclerotic risk. Plant sterols contribute to lowering LDL, while niacin increases HDL and supports lipid metabolism, which is particularly beneficial for those with hypertriglyceridemia and low HDL levels [78,79,80].
- Lipoprotein(a): Elevated lipoprotein(a) is an independent predictor of atherosclerosis and thrombosis. Vitamin C, L-carnitine, and niacin are beneficial for patients with genetically determined elevated lipoprotein(a) levels [81,82].

### 7.2. Additional Lipid Biomarkers for Cardiovascular Risk Assessment

- Apolipoprotein B (ApoB): Elevated ApoB reflects a high concentration of atherogenic lipoproteins, including LDL, and is a more accurate indicator of atherosclerosis risk than total cholesterol. Omega-3 fatty acids and policosanol support ApoB reduction and have anti-atherogenic effects [83].
- Apolipoprotein A1 (ApoA1): Low ApoA1, the main component of HDL, correlates with increased cardiovascular events. Niacin and antioxidants can improve ApoA1 levels and lower cardiovascular risk [84].
- LDL/HDL Ratio: The LDL/HDL ratio provides a clearer assessment of the balance between atherogenic and anti-atherogenic lipoproteins. Omega-3 fatty acids and plant sterols are commonly used to correct high LDL/HDL ratios, optimizing the lipid profile [85].
- Phospholipids: Low phospholipid levels indicate compromised cellular membrane integrity. Phosphatidylcholine and essential fatty acids are recommended for patients with phospholipid deficiencies to support membrane health and lipid metabolism [86].

## 8. Epigenetic, Microbiome, and Immunological Biomarkers for Targeted Nutraceutical Interventions

Epigenetic, microbiome, and immunological biomarkers provide critical insights into environmental, metabolic, and inflammatory influences on a patient’s health. These biomarkers serve as essential tools in precision medicine, identifying hidden deficiencies, optimizing nutritional support, and managing patient health based on unique biological characteristics.

### 8.1. Epigenetic Biomarkers and Their Role in Nutritional Support

Epigenetic biomarkers reflect changes in gene expression influenced by environmental factors such as diet, stress, and toxins, without altering DNA sequences. These markers help clinicians identify potential metabolic dysregulations and predispositions to chronic diseases, allowing for targeted nutritional interventions for prevention and correction.

- DNA Methylation: DNA methylation is a key epigenetic mechanism regulating genes involved in inflammation, metabolism, and aging. Aberrant methylation may indicate deficiencies in B vitamins, particularly folate and B12. Patients with methylation dysregulation may benefit from methylated forms of these vitamins to support optimal epigenetic status [87].
- Histone Modification: Histone acetylation and deacetylation affect DNA packaging density and gene expression. Dysregulated histone modification is associated with increased risks of cancer and neurodegenerative diseases. Supplements such as butyrate, curcumin, and resveratrol can modulate histone modifications, providing preventive effects in these conditions [88].
- MicroRNAs (miRNA): miRNAs regulate the expression of genes related to inflammation, stress, and metabolism. Imbalances in miRNA levels can be markers for targeted support with omega-3 fatty acids and antioxidants like vitamins C and E to reduce oxidative stress and maintain normal miRNA expression [89].

### 8.2. Microbiome Biomarkers and Their Role in Targeted Nutritional Support

Microbiome biomarkers reflect the status of gut microbiota and its impact on metabolic, immune, and inflammatory pathways. Dysbiosis is associated with an increased risk of chronic diseases, including obesity, diabetes, and inflammatory bowel diseases (IBD). Monitoring microbiome biomarkers enables tailored nutritional support to optimize microbiota composition and function.

- Firmicutes/Bacteroidetes Ratio: Altered Firmicutes/Bacteroidetes balance has been linked to obesity and metabolic dysfunction. Increased Firmicutes and decreased Bacteroidetes may indicate an imbalance related to obesity. Probiotics, prebiotics, and dietary fibers are recommended to restore optimal bacterial ratios [90].
- Short-Chain Fatty Acids (SCFAs): SCFAs like butyrate, propionate, and acetate are microbial metabolites essential for regulating inflammation and maintaining gut barrier integrity. Low SCFA levels are associated with inflammatory conditions and irritable bowel syndrome (IBS). Fiber and probiotics like Lactobacillus and Bifidobacterium are recommended to increase SCFA production [91].
- LPS (Lipopolysaccharides): High levels of LPS released by pathogenic bacteria can lead to endotoxemia and systemic inflammation. Patients with elevated LPS benefit from probiotics, antioxidants, and anti-inflammatory nutrients such as omega-3 fatty acids to reduce inflammation and support gut barrier health [92].

### 8.3. Immunological Biomarkers and Their Significance in Nutritional Support

Immunological biomarkers provide insights into the immune system’s condition and inflammatory processes, which are closely linked to risks of infections, autoimmune diseases, and chronic inflammation. Monitoring immunological biomarkers enables precise immune modulation through nutritional support, helping to prevent pathological states.

- C-Reactive Protein (CRP): CRP is a classic marker of systemic inflammation. Elevated CRP indicates acute or chronic inflammation, correlating with an increased risk of cardiovascular disease and type 2 diabetes. Patients with high CRP levels may benefit from anti-inflammatory supplements such as omega-3 fatty acids and curcumin [93,94].
- Interleukin-6 (IL-6): IL-6 is a key inflammatory mediator, with elevated levels linked to autoimmune diseases, obesity, and chronic inflammatory conditions. Omega-3 fatty acids and antioxidants like vitamin C help lower IL-6 levels, providing anti-inflammatory effects [95].
- Tumor Necrosis Factor-alpha (TNF-α): TNF-α is a potent pro-inflammatory cytokine, playing a central role in chronic inflammation and autoimmune diseases. Omega-3 fatty acids, antioxidants, and polyphenols can suppress TNF-α activity, helping to reduce inflammation and autoimmune responses [96].
- Secretory Immunoglobulin A (sIgA): sIgA is the main mucosal immunoglobulin, protecting against pathogens and supporting gut barrier function. Low sIgA levels indicate an increased risk of infections and dysbiosis. Patients with low sIgA benefit from probiotics, B vitamins, and zinc to support mucosal immunity [97].

## 9. The Use of Artificial Intelligence for Comprehensive Biomarker Analysis and Targeted Supplement Prescription

Artificial Intelligence is increasingly being used for comprehensive biomarker analysis and personalized dietary supplement recommendations, enabling the consideration of multiple individual factors and fine-tuning nutritional support with high precision. Traditional supplement prescription methods often fail to account for the complex interactions between genetic, metabolic, lipid, and proteomic profiles, potentially limiting their effectiveness. AI automates the analytical process, prioritizes biomarkers by clinical relevance, and selects the most appropriate supplements, thereby minimizing the risks of unwanted interactions and overdoses. The advantages of AI lie in its ability to conduct complex data analysis, identify intricate biomarker relationships, and provide personalized recommendations that align with each patient’s unique metabolic profile and supplement interactions.

Recent studies confirm the efficacy of AI in personalized medicine. A 48-week study on diabetes management evaluated an AI-powered platform for dietary management in adults with type 2 diabetes. The results showed that groups receiving AI-guided support achieved significant improvements in HbA1c control and greater weight loss compared to traditional methods. The AI platform provided adaptive recommendations based on real-time data and medical feedback, improving blood glucose control and facilitating weight reduction in patients [98].

In cardiovascular health, the GenAIS™ AI-driven supplement prescription tool demonstrated promising results in a randomized controlled trial. Patients receiving AI-guided supplement recommendations showed a 25.3% reduction in LDL-C over a 90-day period compared to a 15.2% reduction in the control group, as well as greater reductions in total cholesterol and triglycerides. This tool utilizes a wide range of supplements, including omega-3 fatty acids, plant sterols, and niacin, customized to each individual’s biomarker profile to optimize lipid management and reduce cardiovascular risks [8].

To help healthcare providers effectively use AI-driven tools, specific platforms and databases are becoming more accessible. Tools like GenAIS™ provide an intuitive interface for inputting patient-specific biomarker data and receiving tailored supplement recommendations based on validated algorithms. Additionally, publicly available resources, such as GeneCards, dbSNP, and the Human Metabolome Database (HMDB), can support biomarker identification and interpretation.

For practical implementation, physicians can use AI platforms in conjunction with patient data collected from electronic health records (EHRs) or wearable devices. These systems analyze biomarker fluctuations in real-time and generate recommendations for supplement types and dosages. Furthermore, emerging guidelines and educational resources are being developed to assist clinicians in integrating AI recommendations into patient care, ensuring alignment with clinical best practices and regulatory standards.

These AI-driven solutions for personalized nutritional support are rapidly evolving, with algorithms becoming more accurate each year, encompassing increasingly comprehensive genetic, metabolic, and biochemical data. In the future, AI technologies are expected to integrate with health monitoring systems, providing physicians with automated, real-time recommendations based on biomarker fluctuations. This approach not only enhances the precision and efficacy of supplement prescriptions but also reduces the risks associated with potential interactions and overdosing, making health support more personalized and safer.

## 10. Practical Recommendations for Physicians on Dietary Supplement Prescription

Assessment of Deficiencies and Biomarker Selection Before recommending dietary supplements, conduct a thorough evaluation, including laboratory tests, to identify nutrient deficiencies and metabolic imbalances. Biomarker selection should consider not only clinical symptoms but also preventive aspects, as biomarker changes can signal early metabolic disruptions before symptoms appear. This approach enables the detection of subclinical deficiencies and allows timely nutritional adjustments.

Individualized Dosage Supplement dosages should be personalized based on the patient’s health status, age, sex, and degree of deficiency. Dosage adjustments should rely on biomarker data and recovery dynamics to prevent excessive accumulation and maximize supplement efficacy.

Consideration of Interactions and Contraindications When recommending supplements, evaluate potential interactions with medications and existing contraindications to minimize adverse effects and complication risks. Special attention should be given to patients on medications with known interactions, such as anticoagulants and antidepressants.

Monitoring and Evaluation of Effectiveness Regular biomarker monitoring is essential to assess the efficacy of the recommended supplements. Periodic analysis enables dosage adjustments and helps determine whether to continue, modify, or discontinue supplementation based on progress and target outcomes.

Patient Education Educate patients on the importance of adherence to the supplement regimen and regular monitoring. Inform them about recommended dosages, optimal intake methods, and potential side effects, emphasizing the need to communicate any adverse effects promptly.

Periodic Review of Supplementation Need Reevaluate the need for supplements periodically, adjusting or discontinuing intake once target biomarker levels are achieved. Transition to maintenance doses or cessation as appropriate to avoid excessive nutrient accumulation and reduce potential risks.

## 11. Limitations and Future Directions

While this review emphasizes the promise of biomarkers in guiding personalized dietary supplementation, significant gaps in the current literature limit the robustness of recommendations. Many studies discussed focus primarily on biomarker variations in clinical patients or their relationship with dietary intake and drug therapies, rather than demonstrating the direct effects of biomarker-guided dietary supplementation on health outcomes. This leaves a critical gap in understanding how such interventions translate into improved clinical or physiological results in broader populations. Furthermore, while correlations between biomarkers and nutritional needs are well-established, there is a lack of robust experimental studies, such as randomized controlled trials (RCTs), that test the causal efficacy of dietary supplementation guided by biomarker analysis.

To address this, it is essential to expand AI training datasets by incorporating diverse and large-scale real-world data from multiple populations and settings. In addition, AI tools must provide transparency features that allow users to assess the reliability of their outputs and the completeness of their underlying training data. Establishing timelines for accumulating sufficient data and ensuring rigorous validation will be critical to improving the robustness and reliability of AI-driven recommendations in clinical practice.

Current research also tends to emphasize clinical settings, limiting the generalizability of findings to non-clinical or healthy populations who might benefit from early preventive interventions. Additionally, long-term studies are scarce, leaving questions about the safety and sustained efficacy of biomarker-driven supplementation unanswered.

Another important limitation lies in the regulatory ambiguity surrounding dietary supplements. According to the FDA definition, supplements are not intended to treat, diagnose, or prevent diseases. However, when combinations of supplements demonstrate significant effects on disease prognosis or risk prevention, their classification becomes less clear. Such combinations may approach the efficacy of medications, raising questions about whether they should be regulated as drugs instead of supplements. This ambiguity represents both a conceptual and regulatory challenge for the field of biomarker-guided supplementation. Future research must address these questions to clarify the distinction between supplements and medications and provide a framework for their appropriate classification and use.

To bridge these gaps, future research should prioritize conducting large-scale RCTs to evaluate the impact of biomarker-guided dietary supplementation on specific health outcomes, including disease prevention, metabolic optimization, and quality of life improvements. Expanding the diversity of study populations to include non-clinical and underrepresented groups will also be essential to ensure broader applicability. Lastly, investigating the long-term safety and effectiveness of these interventions will provide the necessary evidence to establish biomarker-guided supplementation as a core component of personalized nutrition.

This research trajectory will not only strengthen the scientific basis of biomarker-guided dietary supplementation but also pave the way for more precise, safe, and effective strategies in personalized healthcare. Moreover, addressing the regulatory and conceptual challenges of supplement classification will ensure the responsible integration of biomarker-driven approaches into both clinical and consumer health practices.

## 12. Conclusions

Personalized dietary supplementation based on biomarkers offers promising opportunities for precise and effective nutritional support. Biomarkers enable the early detection of deficiencies and metabolic imbalances at a preclinical stage, facilitating timely intervention and prevention. Artificial intelligence enhances this process by analyzing complex biomarker interactions, allowing for tailored adjustments in supplement dosages and combinations that account for the unique characteristics of each patient.

However, significant challenges remain. The reliance of AI-driven tools on incomplete training datasets limits their reliability and generalizability, highlighting the need for more robust and clinically validated algorithms. Additionally, current research often focuses on clinical populations, leaving gaps in applicability for broader and healthier populations who could benefit from preventive approaches. Regulatory ambiguity also complicates the classification of supplements, particularly when their effects approach those of pharmaceutical drugs, creating both conceptual and policy challenges.

The future lies in integrating AI and biomarker analysis into routine clinical practice, empowering clinicians to make evidence-based decisions and improve the outcomes of nutritional interventions. Addressing these limitations will require further research, including large-scale randomized controlled trials, long-term safety studies, and advancements in AI training datasets. By overcoming these challenges, biomarker-guided supplementation can become a cornerstone of personalized medicine, offering an accessible and effective pathway for disease prevention and treatment.
